# Seroprevalence, risk factors and genotyping of *Toxoplasma gondii* in domestic geese (*Anser domestica*) in tropical China

**DOI:** 10.1186/s13071-014-0459-9

**Published:** 2014-10-02

**Authors:** Guang Rong, Han-Lin Zhou, Guan-Yu Hou, Jun-Ming Zhao, Tie-Shan Xu, Song Guan

**Affiliations:** Tropical Crops Genetic Resources Institute, Chinese Academy of Tropical Agricultural Sciences, Ministry of Agriculture, Danzhou, Hainan Province PR China; Scientific and Technical Information Institute, Chinese Academy of Tropical Agricultural Sciences, Danzhou, Hainan Province PR China

**Keywords:** *T. gondii*, Domestic geese, Seroprevalence, Risk factors, Genotype

## Abstract

**Background:**

Little information is available about the seroprevalence of *Toxoplasma gondii* infection in geese (*Anser domestica*) in China. In the present investigation, the seroprevalence, risk factors and genotyping of *T. gondii* in geese were investigated in Hainan province, tropical China.

**Findings:**

A total of 600 serum samples and 150 brain tissue samples were collected from six administrative regions in tropical China, and assayed for *T. gondii* antibodies by Indirect Haemagglutination (IHA) test. Genomic DNA was extracted from the 30 brain tissues of seropositive geese and *T. gondii* B1 gene was amplified using a semi-nested PCR. DNA samples giving positive B1 amplification were then genetically characterized using multi-locus PCR-RFLP. Overall, 17% (95% CI: 14–20) of the animals were positive for *T. gondii* antibodies. Presence of cats in the household (odds ratio, OR 3), hygiene (OR 2.3) and presence of stray cat around the house (OR 2.3) were considered as main risk factors associated with *T. gondii* infection. Of 30 DNA samples, three were positive for the *T. gondii* B1 gene, two showed complete genotyping results. Only one genotype (type II) was identified.

**Conclusions:**

The results of the present survey indicated the presence of *T. gondii* infection in geese in tropical China. Therefore, it is imperative that improved integrated measures be carried out to prevent and control *T. gondii* infection in geese in this province. This is the first report documenting the occurrence of *T. gondii* genotype in geese in China.

## Findings

### Background

*Toxoplasma gondii* is an important zoonotic parasite that infects humans and a wide range of warm-blooded animals, causing toxoplasmosis [1]. It has been estimated that one third of the world population has been infected [2]. The parasite can cause severe disease in the fetus during congenital infection, and can be fatal to immunocomprimised patients such as those with AIDS or organ transplant [3]. To date, toxoplasmosis continues to be a significant public health problem around the world. No commercial vaccines are available, and treatment relies on chemical drugs [4].

*T. gondii* can be acquired by oral ingestion of undercooked meat containing tissue cysts or oocysts from the environment contaminated with infected cat feces, such as soil, foods and water [5]. Goose meat is an important foodstuff worldwide, and humans can acquire infections with *T. gondii* by ingesting undercooked goose meat containing tissue cysts. Surveys of seroprevalence of *T. gondii* infection in geese have been reported throughout the world [6-8], and there have also been surveys of *T. gondii* infection in geese in several provinces of China in recent years [9,10]. The People’s Republic of China (PRC) is one of the largest producers of geese in the world, and Hainan province, tropical China is one of the major geese producers in the PRC. However, there have been no reports of *T. gondii* infection in geese in tropical China. Therefore, investigation of *T. gondii* infection in geese has important implications for the prevention and control of *T. gondii* infection in humans and animals in tropical China.

The objective of the present investigation was to examine the seroprevalence, risk factors and genotyping of *T. gondii* in geese in tropical China. The results should provide base-line data for recommendations with regards to prevention and control of toxoplasmosis in geese in this region and elsewhere.

## Methods

### Ethics statement

The collection of serum samples from geese in the present study was consented by owners of geese, and all geese were handled in strict accordance with good animal practice according to the Animal Ethics Procedures and Guidelines of the People’s Republic of China.

### The study site

The present study was carried out in Hainan province, an island of southern China. This area was geographically separated from Leizhou Peninsula by Qiongzhou strait. Hainan province is situated in the most southern part of China, between the northern latitudes of 3° to 20° and eastern longitudes of 108° to 120°. The climate is the tropical monsoon climate with an average annual temperature of 21–26°C. The annual rainfall ranges from 1000–2600 mm. Hainan province is divided in to 9 administrative regions (cities), with the city of Haikou as its capital.

### Collection and preparation of serum samples

A total of 600 blood samples and 150 brain tissues were collected from six representative administrative regions in Hainan province, tropical China between May 2012 and March 2014 (Table 1). The numbers of geese reared on each farm ranged from 1000 to 5000, approximately. Before sampling, geese were subjected to clinical examination to determine their health status. Information about each goose, such as age, medical history, growth hormones, and weight were collected. Healthy geese were randomly selected for bleeding. Blood samples were then centrifuged at 1,000 *g* for 10 min, and the serum was collected, frozen, and stored at −20°C until assayed.

**Table 1:** **Seroprevalence of** ***Toxoplasma gondii*** **infection in domestic geese in Hainan province, tropical China by indirect hemagglutination assay (IHA)**

**Region**	**Positive no. in different titers**					**No. tested**	**No. positive**	**Prevalence (%)**	**95% CI**
	**1:64**	**1:128**	**1:256**	**1:512**	**1:1024**				
Haikou	4	3	5	2	2	100	16	16	8.8-23.2
Wenchang	6	3	5	3	2	100	19	19	11.3-26.7
Qionghai	3	2	4	3	3	100	15	15	8-22
Danzhou	4	5	6	2	3	100	20	20	12.2-27.8
Dongfang	4	3	5	4	2	100	18	18	10.5-25.5
Sanya	3	2	4	3	2	100	14	14	7.2-20.8
Total	24	18	29	17	14	600	102	17	14-20

An epidemiological questionnaire was fulfilled by farmers who were asked to answer the questionnaire in order to obtain information on the risk factors such as: gender, age of geese, general hygiene of the farm, farm location, cats and dogs in the household, stray cats around the house and outdoor access.

### Serological examination and genetic characterization of *T. gondii* isolates

Antibodies to *T. gondii* were tested by Indirect Hemagglutination antibody (IHA) using a commercially marketed kit (Lanzhou Veterinary Research Institute, Chinese Academy of Agricultural Sciences, Lanzhou, Gansu Province, China). The procedures were used according to the manufacturer’s instructions and previous descriptions [11-14]. Samples that reacted at dilutions of 1:64 or higher were considered positive for *T. gondii* antibodies. Samples that reacted at dilutions of between 1:32 and 1:64 were re-tested, and positive and negative controls were included in each test. The brain tissues of seropositive geese were used for DNA extraction, and then genetic characterization of *T. gondii* isolates from geese was carried out using an established multilocus PCR-RFLP method [15,16]. Six reference *T. gondii* strains were included as the positive controls including GT1, PTG, CTG, MAS, TgCgCa1 and TgCatBr5.

### Statistical analyses

The data were analyzed using PASW Statistics 18 (IBM Corporation, Somers, NY, USA); 95% confidence intervals (CI) are given. The value of *P* < 0.05 differences between levels within factors and interactions were considered to be statistically significant. The risk association was determined by the occurrence probability ratio (odds ratio, OR), and the significance was determined at the 95% confidence interval using the EpiInfo program, version 6.04.

## Results and discussion

The present investigation revealed a high *T. gondii* seroprevalence (17%) in geese, which is higher than those reported in Shenyang and Guangzhou, China [9,10], but was significantly lower than that reported in Germany [6,7]. Differences in *T. gondii* seroprevalence are likely due to differences in animal welfare, climate and husbandry practices. Results of the present and previous investigations [9,10] indicated that *T. gondii* infection was widespread in geese in China. The present survey showed that *T. gondii* seroprevalence in female geese (17.8%) was the higher than those of male geese (16%) (*P* > 0.05); the differences were not statistically significant. The present survey indicated that *T. gondii* seroprevalence in adult geese (20.1%) was the higher than those of young geese (13%), indicating that adult geese have more opportunities for contact with *T. gondii* oocysts. The present survey also showed that *T. gondii* seroprevalence in rural geese (19.1%) was higher than those of suburban geese (12.3%). This interesting feature may be attributed to varied welfare and different living environments of geese.

As far as the domestic or stray cats are concerned, there is no doubt that they play a major role in the epidemiology of toxoplasmosis as a definitive host of *T. gondii*. In the present study, the presence of cats on the goose farms was the main risk factor (domestic cats OR 3; stray cats OR 2.3) (Table 2) for *T. gondii* seroprevalence. Previous epidemiologic observations suggest that cats are essential for the maintenance of *T. gondii* infection in farms through oocyst elimination and contamination of feed and/or water [17]. In addition, our observation is also in accord with the results of a South American risk factor study [18]. The hygiene conditions of geese is another main risk factor associated with *T. gondii* seroprevalence. Logistic regression analysis showed that geese in poor hygiene conditions had a 2.3 times (OR 2.3) (Table 2) higher risk of being seropositive compared to geese in good hygiene conditions.

**Table 2:** **Factors associated with the risk of** ***Toxoplasma gondii*** **in domestic geese in Hainan province, tropical China**

**Risk factor**	**No. examined**	**Negative**	**Positive (%)**	**Odds ratio**	**95% CI**
Gender					
Female	343	282	61 (17.8)	1.1	0.7-1.8
Male	257	216	41 (16)	Reference	
Age					
Adult	338	270	68 (20.1)	1.7	1.1-2.6
Young	262	228	34 (13)	Reference	
Hygiene					
Bad	242	184	58 (24)	2.3	1.5-3.5
Good	358	314	44 (12.3)	Reference	
Area					
Rural	413	334	79 (19.1)	1.7	1-2.8
Suburban	187	164	23 (12.3)	Reference	
Cats in the household					
Yes	331	254	77 (23.3)	3	1.8-4.8
No	269	244	25 (9.3)	Reference	
Dogs in the household					
Yes	413	342	71 (17.2)	1	0.7-1.7
No	187	156	31 (16.6)	Reference	
Stray cat around the house					
Yes	403	321	82 (20.3)	2.3	1.3-3.8
No	197	177	20 (10.2)	Reference	
Outdoor access					
Yes	192	156	36 (18.8)	1.2	0.8-1.9
No	408	342	66 (16.2)	Reference	

Of 30 DNA samples, three were positive for the *T. gondii* B1 gene. Two DNA samples showed complete genotyping results, one from Wenchang and one from Sanya. One positive sample could not be completely genotyped due to low DNA concentration. Only one genotype (type II) was identified from the two positive samples, which were typed at 10 genetic markers, including 9 nuclear loci, i.e., SAG1, 5′-and 3′-SAG2, alternative SAG2, SAG3, GRA6, BTUB, L358, PK1, c22-8 and one on an apicoplast locus Apico. This result provides evidence that type II prevails in the goose population in tropical China. However, the result can only be suggestive because no *T. gondii* strain was isolated.

The modified agglutination test (MAT) is a very sensitive and specific method for *T. gondii* detection in avian species [15,19]. However, IHA is also considered one of the most sensitive and specific serological methods for detecting *T. gondii* antibodies, which has been used extensively in many animals, including pigs, horses, donkeys, yaks, dogs, chickens [11-14,20]. Therefore, the present study used IHA to detect *T. gondii* antibodies in geese utilizing a commercially marketed kit.

Humans can acquire *T. gondii* infection from domestic, wild or companion animals [1-3]. The goose meat is mainly consumed by people and other carnivorous animals in tropical China, and under certain conditions, it is consumed raw or undercooked. The present study indicated that *T. gondii* infection in geese is prevalent. Therefore, infection in geese could be of importance in the epidemiology of toxoplasmosis, and persons in direct or indirect contact with infected geese might be at increased risk. Furthermore, pregnant women working with infected animals are at risk because *T. gondii* is a zoonotic agent that is able to cause abortion in humans [2]. Therefore, further studies are necessary to elucidate the potential impact of *T. gondii* on reproduction of geese.

## Conclusions

The results of the present investigation revealed that *T. gondii* infection in geese is prevalent in Tropical China. Therefore, it is important to execute integrated control strategies and measures to prevent and control *T. gondii* infection in geese in this province. This is the first report documenting the occurrence of *T. gondii* genotype in geese in China.
